# Comparison of the Effectiveness of Nutritional Risk Score-2002 and Royal Free Hospital-Nutritional Prioritizing Tool Nutrition Screening Tools in Liver Cirrhosis

**DOI:** 10.5152/tjg.2025.25632

**Published:** 2025-11-21

**Authors:** Sema Calapkorur, Asli Onur Canaydin, Gulten Can Sezgin, Habibe Sahin

**Affiliations:** 1 Department of Nutrition and Dietetics, Erciyes University Faculty of Health Sciences, Türkiye; 2 Department of Gastroenterology, Erciyes University Faculty of Medicine, Türkiye

**Keywords:** Liver cirrhosis, malnutrition, NRS-2002, nutrition screening tool, RFH-NPT

## Abstract

**Background/Aims:**

Malnutrition is a serious problem in patients with liver cirrhosis; therefore, it is recommended that nutritional screening should be performed regularly with appropriate nutritional screening tools (NSTs). This study aimed to compare the efficacy of the Nutritional Risk Score-2002 (NRS-2002) and Royal Free Hospital-Nutritional Prioritizing Tool (RFH-NPT) NSTs in detecting malnutrition in liver cirrhosis patients.

**Materials and Methods:**

This study was conducted with cirrhotic patients (n = 149). The NRS-2002 and RFH-NPT scales were used to assess the nutritional status of patients, and anthropometric measurements were taken. Biochemical findings of patients were recorded. The Chronic Liver Disease Life Quality Scale 2.0 (CLDLQS 2.0) was used to determine the quality of life.

**Results:**

According to both NSTs, patients with nutritional risk had lower body weight, body mass index, albumin levels, higher C-reactive protein levels, and quality of life scores than patients without nutritional risk (*P* < .05). The sensitivity and specificity of the RFH-NPT were 91.5% and 63.3%, respectively. Albumin was more effective in predicting nutritional risk than other biochemical parameters. The RFH-NPT was found to be more correlated with biochemical parameters than NRS-2002.

**Conclusion:**

TheRFH-NPT is highly effective in detecting malnutrition and correlates with biochemical parameters in cirrhotic patients.

**Main Points**

- Malnutrition is a common and serious problem in patients with liver cirrhosis, requiring regular screening with validated tools.
- The Royal Free Hospital-Nutritional Prioritizing Tool (RFH-NPT) showed higher sensitivity and stronger correlations with biochemical parameters compared to Nutritional Risk Score-2002.
- Albumin was the most effective biochemical marker in predicting nutritional risk.
- Incorporating disease-specific symptoms, RFH-NPT provides a significant advantage in the early diagnosis of malnutrition in cirrhotic patients.

## Introduction

Malnutrition is a common and serious problem in patients with liver cirrhosis and is associated with the progression of liver dysfunction and various complications of liver cirrhosis.u^1^ Early nutritional interventions are essential in patients with cirrhosis to prevent these adverse outcomes, but the lack of a validated rapid nutritional screening tool (NST), fluid retention, and liver dysfunction causes difficulties in interpreting body composition and laboratory results. Therefore, nutritional screening and assessment are not regularly performed in patients with cirrhosis.^1^^,^^2^

Many different NSTs have been developed to assess nutritional status in clinically treated patients. Current Global Leadership Initiative on Malnutrition (GLIM) criteria and ESPEN guidelines recommend the use of the Nutritional Risk Score-2002 (NRS-2002) NST for the assessment of the nutritional status of clinically hospitalized patients.^3^^,^^4^ Although the NRS-2002 includes various components such as body weight loss, body mass index (BMI), and decreased food intake, it does not assess symptoms specific to liver cirrhosis.^5^ Therefore, it is advised that this patient group employs the Royal Free Hospital-Nutritional Prioritizing Tool (RFH-NPT), which was created especially for liver cirrhosis and assesses clinical signs such as fluid retention, edema, and ascites.^1^^,^^4^

This study was planned and conducted to compare the efficacy of NRS-2002 and RFH-NPT in detecting malnutrition in patients with liver cirrhosis.

## Materials and Methods

### Study Plan

This cross-sectional and descriptive study was conducted in the Gastroenterology Clinic of Erciyes University Hospital between December 2022 and 2023. The study population was defined as individuals over 18 years of age who were receiving treatment for liver cirrhosis in the gastroenterology clinic. Patients with loss of consciousness and/or communication problems and patients in the terminal period were not included in the study. The sample size of the study was calculated to be approximately 96 individuals with cirrhosis to obtain a 90% CI based on a previous study.^1^ The study was completed with 149 individuals with cirrhosis (71 males, 78 females) by taking the maximum number of patients who could be reached within the specified period.

Approval of the study was obtained from the Erciyes University Clinical Research Ethics Committee (November 19, 2022, Decision No: 2022/77). In addition, all individuals participating in the study were informed about the study, and their written and verbal consent was obtained. The study was conducted in accordance with the principles of the Declaration of Helsinki.

### Data Collection

Demographic information of patients was obtained via a questionnaire form, and anthropometric measurements [height, body weight] were taken by the researchers following the technique. The BMI [weight (kg)/height (m)^2^] was calculated from the weight and height measurements. Routine biochemical findings (alanine aminotransferase (ALT), aspartate transferase (AST), gamma-glutamyl transferase (GGT), total protein, albumin, C-reactive protein (CRP)) were recorded from patient files.

The Chronic Liver Disease Life Quality Scale 2.0 (CLDLQS 2.0) was used to determine the quality of life of patients. The CLDLQS 2.0 is a disease-specific scale developed to measure the effects of chronic liver disease on quality of life and activities of daily living. The scale consists of 2 parts and includes 24 sub-questions in total. Higher scores (lowest 0 points, highest 96 points) indicate worse quality of life.^6^

The NRS-2002 and RFH-NPT scales were used to assess the nutritional status of patients. The NRS-2002 is a system that scores the deterioration in the nutritional status of patients and the severity of their diseases. Patients with a total score of 3 and above are at risk of malnutrition.^7^

The RFH-NPT is a liver disease-specific NST used to assess nutrition in patients with cirrhosis. This NST calculates the risk of malnutrition in 3 steps. In the first step, the presence of acute alcoholic hepatitis and tube feeding status are questioned. In the second step, fluid retention (presence of peripheral edema/ascites) is questioned. Finally, the scores are summed up to calculate the risk of malnutrition. At low risk (0 points), weekly screening should be repeated; at moderate risk (1 point), nutritional status should be monitored, snacks should be recommended, and weekly screening should be repeated; at high risk (2-7 points), nutritional status should be monitored, snacks should be recommended, nutrition should be encouraged, a nutritional dietitian should be consulted, and weekly screening should be repeated.^8^

### Statistical Analysis

The collected data were analyzed using Statistical Package for Social Sciences (SPSS) version 25.0 (IBM SPSS Corp.; Armonk, NY, USA). The significance level was defined as *P *< .05 for all statistical analyses.

The Kolmogorov–Smirnov test was performed to evaluate the normality of the distribution of numerical variables. Mean and standard deviation were given for normally distributed variables, and median and lower-upper values were given for non-normally distributed variables. The chi-square test for categorical variables, Student’s *t*-test, and Mann–Whitney *U*-test for normally distributed and non-normally distributed continuous variables, respectively, were applied to assess the relationship between groups with or without nutritional risk. The correlation between test results and biochemical parameters was evaluated using the Pearson correlation coefficient. For the RFH-NPT NST, sensitivity, specificity, positive predictive value (PPV) and negative predictive value (NPV) were calculated using the Excel program and expressed as percentages. The ROC curve and area under the curve (AUC) were used to evaluate the performance of biochemical parameters in predicting malnutrition with NRS-2002 and RFH-NPT.

## Results

### Characteristics of Participants

The mean values of various parameters of the participants according to NRS-2002 and RFH-NPT NSTs are shown in Table 1. According to both NSTs, patients at risk of malnutrition had lower body weight, BMI, and albumin levels and higher CRP levels and quality of life scores than patients without malnutrition risk (*P *< .05).

It was found that NRS-2002 and RFH-NPT scores of patients were negatively correlated with serum albumin levels (*r* = −0.203, *P *= .013 and *r* = −0.338, *P *< .001, respectively) and positively correlated with CLDLQS 2.0 scores (*r* = 0.378, *P *< .001 and *r* = 0.488, *P *< .001, respectively). There was also a positive correlation (*r* = 0.593, *P* < .001) between the mean scores of both scales (Table 2).

**Figure 1.**
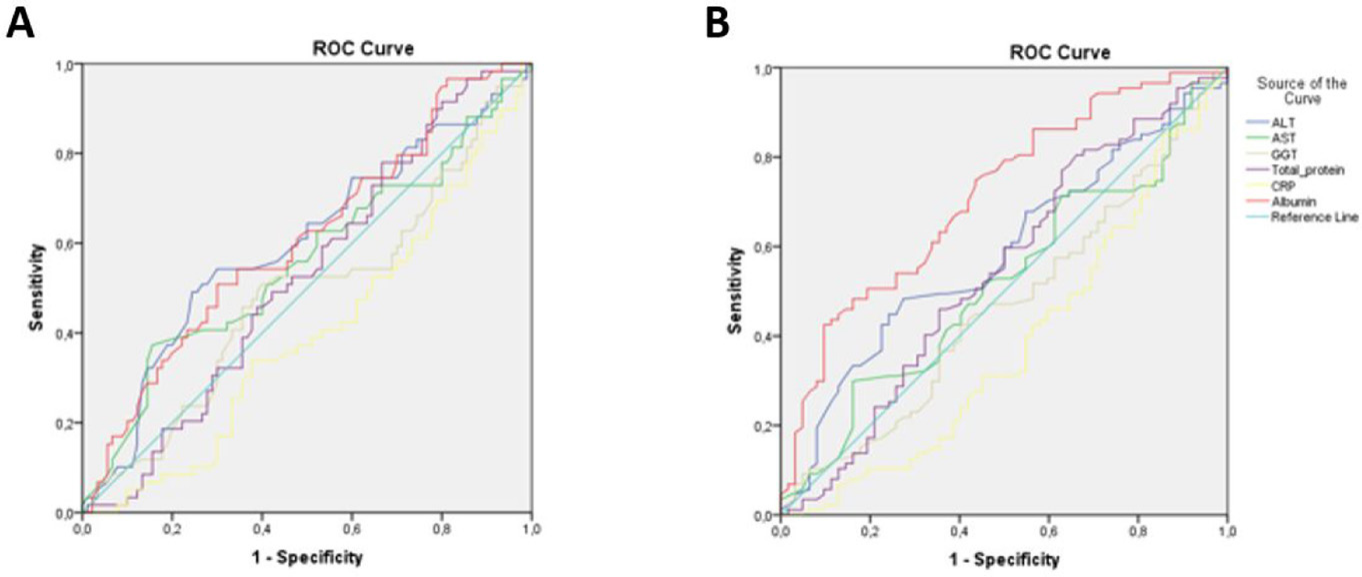
Performance of some biochemical parameters in predicting the results of NSTs used to detect malnutrition. A. NRS-2002, B. RFH-NPT.

The RFH-NPT evaluated according to NRS-2002 had a sensitivity of 91.5%, specificity of 63.3%, PPV of 62.1%, and NPV of 91.9% (not shown in the Figure 1).

The performance of some biochemical parameters in predicting the results of NSTs used to detect malnutrition is shown in Figure 1 with ROC curves. In Figure 1A, albumin (AUC = 0.606, *P* = .029); ALT (AUC = 0.598, *P* = .043), AST (AUC = 0.558, *P* = .231), gamma-glutamyl transferase (GGT) (AUC = 0.4685, *P *= .755), total protein (AUC = 0.525, *P *= .604), and CRP (AUC = 0.396, *P *= .032) were more effective in predicting nutritional risk in NRS-2002. In Figure 1B, albumin (AUC = 0.714, *P *< .001); ALT (AUC = 0.583, *P* = .083), AST (AUC = 0.513, *P* = .709), GGT (AUC = 0.467, *P* = .499), total protein (AUC = 0.543, *P *= .366), and CRP (AUC = 0.382, *P *= .014) were found to be more effective in predicting nutritional risk in RFH-NPT.

## Discussion

Malnutrition is an important problem in patients with liver cirrhosis; therefore, assessment of nutritional status is of great importance.^9^ The NSTs that consider disease-specific symptoms are recommended for the assessment of the nutritional status of patients.^1^ In this context, in the study comparing the efficacy of RFH-NPT and NRS-2002 in detecting malnutrition, albumin levels of patients with malnutrition risk were found to be lower, while CRP levels and quality of life scores were found to be higher in patients without malnutrition risk according to both NSTs (Table 1). Visceral serum proteins such as albumin and prealbumin are used as markers of the nutritional status of patients, and the relationship between malnutrition and albumin has been confirmed in studies conducted in various patient groups.^10^^-^^12^ In addition, high CRP levels are generally reported to be associated with low nutrient intake in hospitalized elderly patients.^13^ Inflammation, which is accepted among the etiologic criteria of malnutrition in GLIM criteria, is associated with malnutrition status in hemodialysis patients.^14^^,^^15^ In this study, the relationship between malnutrition and albumin and CRP levels in cirrhotic patients was confirmed in support of the literature.

Malnutrition is associated with worse clinical outcomes in patients, and this leads to deterioration in the quality of life of patients. In various studies, it has been reported that malnutrition causes deterioration in quality of life.^16^^-^^18^ In the study, it was concluded that patients with malnutrition risk had a worse quality of life according to both NRS-2002 and RFH-NPT (Tables 1 and 2).

In the study, the sensitivity and specificity of RFH-NPT compared to NRS-2002 were analyzed, and the sensitivity and specificity of RFH-NPT were determined as 91.5% and 63.3%, respectively. In another study conducted to validate the RFH-NPT, these values were 97% and 74%, respectively.^19^ These results suggest that the RFH-NPT is a reliable NST to assess the risk of malnutrition in this population. In the study, the NPV value of the RFH-NPT was 91.9%, which confirms this interpretation.

When the relationship between NRS-2002 and RFH-NPT with biochemical parameters was evaluated, the AUC of albumin value was found to be higher in both NSTs. This result shows that albumin is more effective in predicting nutritional risk than other biochemical parameters. In various studies, the relationship between albumin and different NSTs has been demonstrated.^20^^,^^21^ However, no study was found to show its relationship with RFH-NPT. When a comparison was made between the tests in the study, it was determined that RFH-NPT was more correlated with biochemical parameters (especially albumin) in predicting nutritional status (Figure 1A and B). Although there are studies in the literature evaluating the efficacy of RFH-NPT in determining malnutrition in cirrhotic patients^1^^,^^20^ no study examining its relationship with biochemical parameters has been found. It is thought that this study will make an important contribution to the field in this sense.

In this study, it was determined that the efficiency of RFH-NPT in determining malnutrition and its relationship with biochemical parameters was high in cirrhotic patients. In this context, it can be said that the use of RFH-NPT, which also considers disease-specific symptoms in the evaluation of nutritional status in patients with liver cirrhosis, is important and necessary in diagnosing malnutrition at an early stage.

### Study Limitations

This study has several limitations. It was conducted as a single-center, cross-sectional study, which restricts the generalizability of the findings and prevents evaluation of long-term outcomes. Dietary intake and physical activity were not comprehensively assessed, which may have influenced nutritional and biochemical results. Other anthropometric measurements such as neck or mid-arm circumference were not included, limiting a more detailed nutritional assessment. In addition, the study did not compare the screening tools with a gold standard such as Subjective Global Assessment (SGA) or GLIM criteria. Despite these limitations, the study contributes by increasing awareness of the clinical use of the RFH-NPT tool.

## Figures and Tables

**Table 1.** Characteristics of the Participants

	**NRS-2002**			**RFH-NPT**		
	**No/Low Risk**	**High Risk**	***P***	**Low Risk**	**Moderate/High Risk**	***P***
Age (years)	61.50 ± 11.59	66.54 ± 11.11	.009*^c^	63.29 ± 12.27	64.00 (33.00-88.00)	.787^b^
Gender						
Male, n (%)	47 (52.2)	24 (40.7)	.168^a^	33 (53.2)	38 (43.7)	.250^a^
Female, n (%)	43 (47.8)	35 (59.3)		29 (46.8)	49 (56.3)	
Height (cm)	164.77 ± 10.97	162.00 (147.00-178.00)	.148^b^	164.73 ± 11.76	162.00 (149.00-184.00)	.219^b^
Weight (kg)	78.63 ± 15.53	68.00 (45.00-124.00)	.010^*^ ^b^	80.55 ± 15.91	72.72 ± 16.66	**.005** ^*^^c^
BMI (kg/m^2^**)**	27.87 (20.03-45.72)	26.64 (17.15-48.44)	**.033** ^*^^b^	29.05 (21.86-45.72)	26.64 (17.15-48.44)	.006^*^ ^b^
ALT (U/L)	26.50 (5.00-646.00)	20.00 (3.00-992.00)	**.043** ^*^^b^	26.00 (5.00-269.00)	23.00 (3.00-992.00)	.083^b^
AST (U/L)	43.00 (12.00-221.00)	35.00 (11.00-1016.00)	.231^b^	41.50 (13.00-221.00)	37.00 (11.00-1016.00)	.709 ^b^
GGT (U/L)	70.00 (6.00-547.00)	61.00 (10.00-597.00)	.755^b^	67.50 (6.00-547.00)	74.00 (9.00-597.00)	.499^b^
Total protein (g/dL)	6.38 ± 1.00	6.31 ± 0.80	.620^c^	6.42 ± 1.00	6.31 ± 0.88	.478^c^
Albumin (g/dL)	3.38 ± 0.79	3.12 ± 0.65	**.036** ^*^^c^	3.60 ± 0.70	3.04 ± 0.70	**<.001** ^*^^c^
CRP (mg/L)	7.41 (0.04-213.50)	11.40 (0.85-127.00)	**.032** ^*^^b^	6.21 (0.04-213.50)	11.30 (0.50-207.00)	**.014** ^*^^b^
CLDLQS 2.0 score	49.82 ± 16.99	61.07 ± 15.29	<.001^*^ ^c^	41.00 (24.00-87.00)	60.83 ± 14.88	**<.001** ^*^^b^

X ± SD values were given for quantitative data showing normal distribution and “Median (Lower-Upper)” values were given for data not showing normal distribution.

*P < .05.

^a^Pearson Chi-square test.

^b^Mann–Whitney *U*-test.

^c^Independent sample t-test.

ALT, alanine transaminase; AST, aspartate aminotransferase; CLDLQS 2.0, Chronic Liver Disease Life Quality Scale 2.0; CRP, C-reactive protein; GGT, gamma glutamyl transferase; NRS-2002, Nutrition Risk Screening 2002; RFH-NPT, Royal Free Hospital-Nutritional Prioritizing Tool.

**Table 2.** Correlation of Participants’ NRS-2002 and RFH-NPT Scores with Some Parameters

		**Male (n = 71)**		**Female (n = 78)**		**Total (n = 149)**	
		***r***	***P***	***r***	***P***	***r***	***P***
NRS-2002	RFH-NPT	0.580	**<.001**	0.601	**<.001**	0.593	**<.001**
	CLDLQS 2.0 score	0.456	**<.001**	0.288	**.010**	0.378	**<.001**
	ALT	−0.030	.804	0.123	.281	0.046	.576
	AST	−0.006	.961	0.119	.297	0.076	.354
	GGT	0.060	.622	0.007	.954	0.011	.890
	Total protein	−0.003	.979	−0.071	.537	−0.042	.610
	Albumin	−0.150	.213	−0.258	**.023**	−0.203	**.013**
	CRP	0.165	.170	0.115	.316	0.133	.107
RFH-NPT	CLDLQS 2.0 score	0.567	**<.001**	0.410	**<.001**	0.488	**<.001**
	ALT	0.117	.331	0.102	.376	0.100	.223
	AST	0.065	.589	0.104	.366	0.084	.310
	GGT	0.089	.463	0.059	.611	0.056	.498
	Total protein	−0.112	.350	−0.018	.875	−0.063	.445
	Albumin	−0.321	**.006**	−0.357	**.001**	−0.338	**<.001**
	CRP	0.224	.061	−0.018	.879	0.101	.219

ALT, alanine transaminase; AST, aspartate aminotransferase; CLDLQS 2.0, Chronic Liver Disease Life Quality Scale 2.0; CRP, C-reactive protein; GGT, gamma glutamyl transferase; NRS-2002, Nutrition Risk Screening 2002; RFH-NPT, Royal Free Hospital-Nutritional Prioritizing Tool.

## Data Availability

The data that support the findings of this study are available on request from the corresponding author.
